# Exploring the Relationship Between Hepatitis C Virus Infection and Prostate Cancer Risk: A National Health and Nutrition Examination Survey Analysis

**DOI:** 10.7759/cureus.54523

**Published:** 2024-02-20

**Authors:** Marc Ganz, Christopher Alessandro, Menachem Jacobs, Yehuda Gejerman, Daniel Miller, Frederick Okoye, Scott Jamieson, Andrew Winer

**Affiliations:** 1 Public Health Sciences, State University of New York Downstate Health Sciences University, New York City, USA; 2 Internal Medicine, New York Medical College, Valhalla, USA; 3 Internal Medicine, Icahn School of Medicine at Mount Sinai, Queens Hospital Center, New York City, USA; 4 Urology, State University of New York Downstate Health Sciences University, New York City, USA

**Keywords:** risk, hcv, nhanes, prostate cancer, hepatitis c

## Abstract

Introduction

Prostate cancer and hepatitis C virus (HCV) infection stand as notable worldwide health issues. Investigating the connection between HCV infection and the risk of prostate cancer remains an ongoing endeavor, complicated by contradictory findings in prior research. It is imperative to comprehend this potential relationship in order to enhance strategies for prevention and treatment. This paper seeks to delve into the association between HCV infection and prostate cancer by analyzing data from the National Health and Nutrition Examination Survey (NHANES), a comprehensive cross-section of the US population.

Methods

Information extracted from the NHANES dataset encompassed the period spanning from March 2017 to March 2020, with a focus on the "medical conditions" and "hepatitis" segments. Employing logistic regression analysis, we aimed to discern the connection between HCV infection and the prior occurrence of prostate cancer. This analysis was conducted while factoring in variables such as weight, hypertension, hyperlipidemia, race, educational level, and marital status to ensure the accuracy of the findings. The results of this examination yielded adjusted odds ratios (OR), coefficients of association (B), and corresponding confidence intervals (CI).

Results

The outcomes derived from the comprehensive multivariate logistic regression analysis, utilizing NHANES data, indicated an absence of a statistically noteworthy correlation between HCV infection and the probability of prostate cancer occurrence. While accounting for diverse variables like weight, hypertension, hyperlipidemia, race, educational level, and marital status, no substantial relationship was observed between HCV infection and the risk of prostate cancer. These results are consistent with earlier investigations that similarly struggled to establish a definitive connection between HCV infection and the incidence of prostate cancer.

Conclusion

Drawing from NHANES data, this study indicates the absence of a substantial link between HCV infection and the incidence of prostate cancer. The divergent findings observed in prior research accentuate the intricate nature of the connection between HCV infection and prostate cancer. Future investigations should encompass more extensive sample sizes, prospective frameworks, and a meticulous assessment of potential variables that might confound the results. Furthermore, it is important to examine the potential protective impact of HCV infection due to antiviral interventions and its effect on the associated risk of prostate cancer. Such endeavors would offer valuable insights for individuals grappling with these health challenges.

## Introduction

Prostate cancer holds the position of being the second most frequently diagnosed cancer in men across the world, with approximately 1.4 million new cases recorded in 2020 [1]. Concurrently, hepatitis C virus (HCV) infection poses a global public health challenge, with an estimated 71 million individuals grappling with persistent HCV infection [2]. The prominence of both prostate cancer and HCV infection on a global scale is unmistakable. Despite the substantial research conducted on prostate cancer, the relationship between HCV infection and the risk of prostate cancer remains a field under scrutiny. Unraveling the potential connection between HCV infection and prostate cancer stands to offer valuable insights into the intricate mechanisms governing prostate cancer development, potentially paving the way for enhanced preventive and management strategies. Thus, it is imperative to thoroughly investigate the interplay between HCV infection and prostate cancer, not only to cater to the healthcare necessities of individuals affected by these conditions but also to formulate precisely targeted measures for prevention and treatment.

Numerous investigations have documented a noteworthy observation: individuals afflicted by chronic HCV infection appear to face an elevated likelihood of developing prostate cancer when juxtaposed with those who lack such an infection [3-5]. The potential mechanism underlying this intricate connection between HCV infection and prostate cancer involves the notion of chronic inflammation. HCV, in its chronic state, has the capacity to incite persistent inflammation within not only the liver but also various other organs, precipitating tissue impairments and a spectrum of health complications [6]. It is plausible that this ongoing inflammation could lay the groundwork for the emergence of diverse health issues, prostate cancer among them [7]. This supposition posits chronic inflammation induced by HCV infection as a potential catalyst in the intricate interplay leading to prostate cancer development.

Nonetheless, even with studies showcasing an augmented risk, the correlation between HCV infection and prostate cancer remains a subject of contention. Certain investigations have encountered challenges in solidifying a definitive link between HCV infection and prostate cancer [8,9], hinting at the potential involvement of other influencing elements or confounding variables within the observed connections. Acknowledging that the progression of prostate cancer entails a multifaceted process molded by diverse genetic, environmental, and lifestyle components, it is pivotal to recognize that the role of HCV infection in this risk interplay might be intricately shaped by these multifarious interactions.

Interestingly, in contrast to the studies indicating an increased risk, there is emerging evidence suggesting a potential protective effect of HCV infection against prostate cancer. Some studies have reported a lower incidence of prostate cancer in individuals with HCV infection compared to those without HCV infection [10,11].

Interestingly, diverging from the studies that point towards an escalated risk, recent evidence is surfacing that proposes a conceivable safeguarding influence of HCV infection against prostate cancer. A handful of studies have brought to light a diminished occurrence of prostate cancer among individuals with HCV infection when contrasted with those without infection [10,11]. These findings spark intriguing questions about the fundamental mechanisms at play and underscore the intricate nature characterizing the interrelation between HCV infection and the risk of prostate cancer. The role of antiviral treatment for HCV and its potential influence on the risk of prostate cancer merits closer examination. Considering the plausible connection between HCV infection and prostate cancer, there exists a need for expanded research efforts to gain a more comprehensive understanding of the dynamic between these two conditions.

The objective of this paper is to delve into the correlation between HCV infection and prostate cancer through the utilization of data sourced from the National Health and Nutrition Examination Survey (NHANES). Within the NHANES database lies a meticulously curated cross-section that represents the entirety of the US population, encompassing vital insights concerning both HCV infection and occurrences of prostate cancer. By examining the connection between HCV infection and prostate cancer via NHANES data, the intention of this study is to contribute to the existing body of knowledge and enhance the insight into potential risk factors associated with prostate cancer.

## Materials and methods

Data collection

Data collection centered on the CDC's National Health and Nutrition Examination Survey (NHANES) dataset, encompassing questionnaire responses recorded from March 2017 to March 2020, specifically categorized under the sections labeled "medical conditions" and "hepatitis." The research protocol was granted approval by the Physician's Journal of Medicine Review Board located in Queens, New York, United States (approval number: 2205F007).

Variables considered

To explore the potential linkage between hepatitis C and the history of prostate cancer, a comprehensive logistic regression analysis was employed. This analysis was adjusted to account for influential variables, including weight, hypertension, hyperlipidemia, race, educational level, and marital status. Within the framework of this study, the dependent variable of interest was the historical occurrence of prostate cancer. This was evaluated by affirming a "yes" response to the survey query "Have you ever been told you had cancer or malignancy?" and indicating "prostate" as the response to the subsequent query "What kind was it?". The independent variable under consideration was hepatitis C, discerned through an affirmative answer to the question "Has a doctor or other health professional ever informed you that you have hepatitis C?". Additionally, covariates such as BMI, age, race, educational level, and marital status were subjected to analysis.

Statistical analysis

The data was organized and recorded on a spreadsheet, which was used for further analysis of the data. To evaluate the relationship between the predictor and outcome variables detailed earlier, a multivariate logistic regression approach was utilized to investigate the association between hepatitis C and prostate cancer. The analysis encompassed a range of variables, including BMI, age, race, educational level, and marital status. Within this analytical framework, adjusted odds ratios (OR), coefficients of association (B), and corresponding confidence intervals (CI) were computed, affording a comprehensive insight into the interrelation. A p-value of 0.05 was used as a cutoff for statistical significance. All statistical analyses were conducted using SPSS Statistics for Windows version 28.0 (Released 2021; IBM Inc., Armonk, New York).

## Results

Data (Table 1,2) showed a cohort comprising of 61 individuals with a history of prostate cancer and 611 individuals devoid of such a history. Among those who had experienced prostate cancer, the racial distribution was as follows: three (4.92%) were of Hispanic origin, nine (14.06%) were Mexican American, 84 (14.00%) were other Hispanic, 46 (24.60%) were White, four (8.16%) were Black, and four(8.89%) were Asian. Pertaining to educational attainment, the distribution within the prostate cancer group was as follows: six (10.71%) with less than a ninth-grade education, 14 (13.59%) with a 9-11th grade education, 42 (18.69%) high school graduates or equivalent, 47 (13.65%) with some college or an associate degree, and 40 (14.61%) with a college degree or above. Within the category of marital status, those with a history of prostate cancer demonstrated 94 (16.96%) as married, 49 (13.58%) as widowed/divorced/separated, and seven (7.87%) as never having been married.

**Table 1:** Demographics of patients with prostate cancer

Variable	History of prostate cancer N (%)	No history of prostate cancer N (%)
Race		
Hispanic origin	3 (4.92%)	58 (95.08%)
Mexican American	9 (14.06%)	55 (85.94%)
Other Hispanic	84 (14.00%)	516 (86.00%)
White	46 (24.60%)	141 (75.40%)
Black	4 (8.16%)	45 (91.84%)
Asian	4 (8.89%)	41 (91.11%)
Education level		
Less than 9th grade	6 (10.71%)	50 (89.29%)
9-11th grade	14 (13.59%)	89 (86.41%)
High school graduate/GED or equivalent	42 (18.69%)	183 (81.31%)
Some college/AA degree	47 (13.65%)	297 (86.35%)
College graduate or above	40 (14.61%)	234 (85.39%)
Marital status		
Married	94 (16.96%)	460 (83.04%)
Widowed/divorced/separated	49 (13.58%)	312 (86.42%)
Never married	7 (7.87%)	82 (92.13%)

**Table 2:** Correlation coefficients of variables and prostate cancer Hep C - hepatitis C; BMI - body mass index; SBP - systolic blood pressure; DBP - diastolic blood pressure; LDL - low-density lipoprotein

Variable	Odds ratio	B	Lower 95% CI	Upper 95% CI	p-value
Hep C	21.458	21.458	-87520.918	87563.834	1
Age	0.069	0.069	0.021	0.118	0.005
BMI	-0.049	-0.049	-0.113	0.014	0.129
SBP	3.53E-05	3.53E-05	0	0	0.819
DBP	-7.07E-05	-7.07E-05	0	0	0.639
LDL	4.00E-05	4.00E-05	-5.28E-05	0	0.398
Race	0.521	0.521	-0.296	1.337	0.211
Education level	-0.125	-0.125	-0.44	0.19	0.437
Marriage	0.78	0.78	-0.051	1.612	0.066

The analysis of odds ratios unveiled a lack of noteworthy association between the hepatitis C virus (HCV) and prostate cancer (p-value of 1.0). On the other hand, age exhibited a significant correlation with prostate cancer (p-value = 0.005), indicating that each incremental increase in age was linked with an elevation in the odds of experiencing prostate cancer. Conversely, body mass index (BMI), systolic blood pressure (SBP), diastolic blood pressure (DBP), low-density lipoprotein (LDL) cholesterol, race, education level, and marital status exhibited no substantial associations with prostate cancer.

## Discussion

The intricate interplay between HCV infection and prostate cancer presents a compelling subject for exploration in light of the global significance of these health concerns. Both diseases contribute substantially to the global health landscape. This study aimed to delve into the potential association between HCV infection and prostate cancer, leveraging the rich dataset provided by the NHANES.

The observed inconsistency in the reported relationship between HCV infection and prostate cancer underscores the complexity of this nexus. Notably, existing research has yielded divergent results, with some studies suggesting an augmented risk of prostate cancer among patients with chronic HCV infection or no correlation at all [3-5], while others point toward a potential protective effect [10,11]. The mechanism underpinning the connection between HCV infection and prostate cancer, which hints at chronic inflammation as a plausible causative factor [6], adds depth to the exploration of this relationship.

The present study aims to contribute to the current body of literature by expanding upon this intricate web of associations. Utilizing the NHANES database, a nationally representative sample of the US population, we examined the relationship between HCV infection and prostate cancer. Employing multivariate logistic regression analysis, adjusted for a range of influential variables, the results indicated a lack of statistically significant association between HCV infection and the likelihood of experiencing prostate cancer. These findings resonate with previous studies that also failed to establish a definitive link between the two conditions [8,9].

The current study comes with a set of inherent limitations that warrant recognition. The cross-sectional design in the NHANES dataset inherently impedes the establishment of a causal link between HCV infection and prostate cancer. Furthermore, the dependence on self-reported data for diagnosing HCV infection and prostate cancer introduces the potential for recall bias and inadvertent misclassification. Moreover, the intricate and multifaceted nature of prostate cancer, coupled with the existence of numerous confounding variables, challenges the feasibility of entirely isolating dependent variables concerning independent variables despite the implementation of controls for various common factors.

While this retrospective study was found not to be clinically significant, there still remain many understudied factors. The immune system is crucial in helping prevent cancers [12,13]. Therefore, it would be logical that any immune modulating factor may alter the risks of developing cancers. HCV was found to cause circulating cryoglobulins, which altered the immune system's functioning and caused extrahepatic effects [14]. Part of the reason why the elderly are more predisposed to cancer is due to the breakdown of the immune system [15]. More research is necessary in this area. 

## Conclusions

The findings derived from this investigation lend weight to the absence of any substantial association between HCV infection and the risk of prostate cancer. In contrast to the varying outcomes of previous studies, the present analysis, grounded in NHANES data, has not unveiled a statistically significant connection between HCV infection and the likelihood of developing prostate cancer. This study thus enhances the ongoing discourse on the intricate interplay between HCV infection and the risk of prostate cancer. The divergent landscape of research findings necessitates a deeper exploration, one that encompasses larger sample sizes, prospective study designs, and a holistic consideration of potential confounding variables. By delving into these complex relationships, a more nuanced understanding can be gleaned, facilitating the development of enhanced prevention and management strategies for both HCV infection and prostate cancer.

In light of the intriguing possibility of HCV infection exerting a protective influence against prostate cancer, there is merit in unearthing the underlying mechanisms that govern this relationship, including the interplay of antiviral treatments for HCV in the context of prostate cancer risk. Such insights stand to have far-reaching implications, not only for advancing our comprehension of these complex health concerns but also for refining the healthcare approaches that cater to the diverse needs of those affected by these conditions.
